# Employee volunteering, knowledge integration, and career sustainability: the moderating role of competitive orientation

**DOI:** 10.3389/fpsyg.2026.1876278

**Published:** 2026-07-28

**Authors:** Lei Gong, Jiayu Li, Xiaoyun Han, Lei Tang, Han Qiu

**Affiliations:** Alibaba Business School, Hangzhou Normal University, Hangzhou, China

**Keywords:** career sustainability, competitive orientation, employee volunteering, knowledge integration, social learning theory

## Abstract

**Purpose:**

In the context of digital transformation and intensified global competition, career sustainability has become a core issue for both organizational and individual development. This study investigates how employee volunteering influences career sustainability by examining the mediating role of knowledge integration and the moderating role of competitive orientation.

**Design/Methodology/Approach:**

Based on social learning theory, a three-wave time-lagged survey was conducted among 293 employees from Chinese internet companies. The study employed hierarchical regression analysis, confirmatory factor analysis, and moderated mediation analysis to test the hypothesized relationships.

**Findings:**

The results show that employee volunteering positively affects career sustainability through the mediating effect of knowledge integration, and that this effect is weakened by higher levels of competitive orientation.

**Originality:**

This study develops an integrated framework linking company-supported employee volunteering to career sustainability through knowledge integration, thereby extending research on how prosocial activities may contribute to sustainable careers. It further extends research on competitive orientation by identifying it as a boundary condition, thus offering actionable insights into balancing competition and collaboration in organizational contexts.

**Practical implications:**

Our research findings provide a better understanding of the mechanisms linking volunteering and career development and offer managers and policymakers useful guidance on promoting employees’ long-term development through volunteering initiatives.

## Introduction

The rapid advancement of technology coupled with socioeconomic uncertainties has led to continuous evolution in the professional landscape, making career sustainability an issue of growing concern for individuals, organizations, and society. To adapt to rapidly changing environments and technological developments, both individuals and organizations are in critical need of sustainable career development (Lawrence et al., 2015). Career sustainability refers to an individual’s enduring career experiences over time (Van Der Heijden and De Vos, 2015). Career sustainability reflects individuals’ long-term employability, well-being, and capacity to adapt across changing work contexts (Bozionelos et al., 2020; Chin et al., 2022; De Vos et al., 2020; De Vos and Van Der Heijden, 2017). Consequently, career sustainability yields integrative benefits by fostering individual lifelong work competence and wellbeing, sustaining organizational performance and adaptive capacity, and enhancing societal economic stability and labor market efficiency.

Research on how to enhance career sustainability within organizational contexts—such as effective interventions or strategies to promote its development—is still in the early stages (De Vos et al., 2020; Kong et al., 2023), with current scholarly efforts still predominantly focused on identifying influencing factors (e.g., organizational support, individual career competencies) rather than actionable improvement pathways. Prior research has shown that career sustainability is linked to factors such as career competencies (Blokker et al., 2019), human resource practices (Tordera et al., 2020), career adaptability (Nakra and Kashyap, 2023), and career shocks (Pak et al., 2021). These studies suggest that employees’ physical health, well-being, and productivity are crucial for their career sustainability (De Vos et al., 2020; Kong et al., 2023). However, existing studies have paid limited attention to organization-supported prosocial activities that go beyond employees’ formal job tasks, particularly the mechanisms through which employee volunteering influences career sustainability.

With enterprises placing increasing emphasis on social responsibility, employee volunteering is gradually becoming a significant topic of discussion. This trend is particularly salient among internet companies. Rather than referring only to corporate social responsibility initiatives at the organizational level, employee volunteering emphasizes employees’ active participation in company-organized or company-supported social service and public welfare activities (Rodell et al., 2016). Prior research suggests that such activities not only reflect corporate values but also benefit employees by enhancing fulfillment, engagement, and job performance (Aguinis and Glavas, 2012; Do Paço and Cláudia Nave, 2013; Rodell et al., 2016, 2017). Moreover, employee volunteering has been linked to positive organizational outcomes, including employee retention (De Gilder et al., 2005; Kim et al., 2010; Peterson, 2004). Therefore, this study aims to specifically explore the impact of employee volunteering on career sustainability and investigate the underlying mechanisms.

This study introduces knowledge integration as a key mediating mechanism. Grounded in social learning theory, employee volunteering provides opportunities for observational learning. During volunteering activities, employees are exposed to diverse scenarios and cross-domain knowledge. Through observation, interaction, and feedback, they can systematically integrate new and existing knowledge and skills (Gill, 2023; Toner and Martins, 2022). The knowledge acquired thereby helps employees address complex career challenges and enhances their career adaptability and sustainability (Baartman and De Bruijn, 2011; Khaksar et al., 2020). Therefore, we propose that employee volunteering ultimately sustains long-term career development by enhancing their knowledge integration capacity.

Furthermore, considering the reality of China’s fiercely competitive internet industry, employees’ competitive orientation may play a significant role in this process. Social learning theory emphasizes how environmental factors constrain learning outcomes. A high competitive orientation may lead employees to focus excessively on social comparisons and advantage-seeking, thereby reducing their willingness to cooperate and engage in knowledge-sharing behaviors (Zhang Y. et al., 2022), diminishing the learning motivation to acquire and integrate knowledge gained through volunteering activities (Jones et al., 2017). Thus, competitive orientation may weaken the positive pathway through which employee volunteering influences career sustainability via knowledge integration.

This study makes three theoretical contributions: First, this study extends the employee volunteering literature by examining employee volunteering as an antecedent of career sustainability, thereby responding to scholars’ call for insufficient exploration of the “actionable pathways” in the formation mechanism of career sustainability (De Vos et al., 2020). Second, it deepens the understanding of the formation mechanisms of career sustainability by revealing the intrinsic relationship between employee volunteering and career sustainability. By identifying knowledge integration as a mediating mechanism, this study helps explain how volunteering experiences are transformed into career-related resources, thereby contributing to the emerging discussion on the intersections among work, learning, and volunteering (Biermann et al., 2024). Third, by verifying the moderating role of competitive orientation, this study uncovers the potential suppressive effect of competition on informal learning and career development, extending existing research. In informal learning contexts such as volunteering, which emphasize collaboration and sharing, a strong competitive motivation can instead create pressure that inhibits learning and integration effectiveness.

## Theoretical background and hypotheses development

### Employee volunteering

Existing research demonstrates that employee volunteering plays a significant positive role in organizational and individual development. For instance, employee volunteering positively influences organizational attitudes and behaviors (Howard and Serviss, 2022). Furthermore, employee volunteering contributes to the enhancement of various individual competencies, including innovative capabilities, work engagement, and interpersonal skills (Booth et al., 2009; Caligiuri et al., 2013; Pasricha et al., 2023; Rodell et al., 2017). The personal outcomes of employee volunteering encompass need fulfillment and overall well-being. Empirical evidence indicates that employees can satisfy diverse personal needs through volunteering, deriving a sense of accomplishment and believing in their potential for growth and development through such experiences (Booth et al., 2009; Caligiuri et al., 2013).

However, the existing studies are geographically uneven, mainly concentrated in developed countries, and there are relatively few studies on employee volunteering in the context of developing countries (Howard and Serviss, 2022). Furthermore, most of the current studies focus on the positive impact of employee volunteering on individual capabilities and organizational outcomes, while paying less attention to the employees’ own experiences, and insufficient attention has been paid to employee volunteering as a form of learning for continuous professional development (Biermann et al., 2024). Moreover, the relationship between employee volunteering and career sustainability has not yet been studied and requires further exploration.

### Employee volunteering and knowledge integration

Knowledge integration is defined as “the process of synthesizing individual expertise into systematic knowledge tailored to specific contexts” (Alavi and Tiwana, 2002), and involves combining, applying, and assimilating diverse specialized knowledge (Bhandar et al., 2007). Knowledge integration involves filtering and structuring relevant information, aligning it with prior knowledge, and leveraging diverse skills and expertise to absorb, recombine, and build upon knowledge for goal attainment (Alavi and Tiwana, 2002; Dussart et al., 2021). Previous research has shown that employee volunteering helps volunteers acquire knowledge, skills, and attitudes beneficial to their careers (Puiu and Udriștioiu, 2023) and promotes knowledge sharing (Toner and Martins, 2022). The acquisition and sharing of knowledge provide the information sources necessary for knowledge integration, broadening one’s knowledge horizons.

Moreover, social learning theory posits that individuals learn not only through their own experiences but also by observing the behaviors and outcomes of others (Bandura, 1977). According to social learning theory, when employees engage in volunteering, they are often exposed to different work contexts and cross-disciplinary knowledge, interacting with individuals from diverse backgrounds, observing others’ work styles, problem-solving approaches, and the sharing of experiences (Toner and Martins, 2022). These processes help employees acquire new knowledge, which contributes to knowledge integration.

In addition, social learning theory emphasizes that individuals adjust their behavior based on feedback gained from interactions with others. During employee volunteering, employees typically interact with other volunteers, organizational members, and people in the community, often receiving positive feedback through these interactions (Boezeman and Ellemers, 2008; Gill, 2023). This feedback helps employees further develop their ability to integrate knowledge from multiple domains.

Based on these, we propose the following hypothesis:

*H1*: Employee volunteering has a positive impact on knowledge integration.

### Knowledge integration and career sustainability

The continuity of career experiences gradually unfolds over time, bringing not only a sense of meaning to the individual employee but also many benefits to the organization (Chin et al., 2022). In this context, numerous studies have shown that both situational and individual factors can influence career sustainability. Empirical research has examined various contextual factors affecting career sustainability, including inclusive leadership (Fang et al., 2021), organizational training (Bozionelos et al., 2020), career shock events (Pak et al., 2021) and sustainable HRM (Nakra et al., 2024). Regarding individual factors, prior studies have primarily concentrated on personality traits such as openness (Bozionelos et al., 2020), competencies (Zhang W. et al., 2022), and attitudinal-cognitive orientations (Xu et al., 2020) as determinants of career sustainability. However, current research still lacks empirical examination of the linkage between individual-level knowledge integration and career sustainability.

With the continuous advancement of AI technologies and evolving business models, technological threats to the labor market have become increasingly pronounced (Decker et al., 2017), necessitating further development and adaptation across all employee categories (Margherita and Braccini, 2021). In the contemporary era, one of the most significant challenges employees face regarding career sustainability involves securing their employment positions (Chin et al., 2019; Larivière et al., 2017; Xu et al., 2018). Numerous studies have identified knowledge as a crucial factor in achieving career sustainability. For instance, employees can leverage unique knowledge to attain career sustainability, thereby differentiating themselves from others (Renn, 2020). Simultaneously, Van Den Groenendaal et al. (2022) emphasize that employees seeking to maintain their positions must diligently work to expand and develop their knowledge daily, applying it effectively to assigned tasks.

From the perspective of social learning theory, the value of knowledge integration for career sustainability lies in its role in transforming learning experiences into adaptive career behavior. Social learning theory emphasizes that individuals learn through observation, interaction, feedback, and the adjustment of subsequent behavior (Bandura, 1985; Xiao and Mao, 2022). Knowledge integration refers not only to the fusion of interdisciplinary knowledge but also to the application and adaptation of acquired knowledge in dynamic work environments (Acharya et al., 2022; Li et al., 2023), helping employees better adapt to new career challenges and changing work demands, thereby enhancing their career adaptability. Prior research has shown that knowledge integration can transform learning-related conditions into work-related outcomes (Li et al., 2023), and that knowledge-based capabilities contribute to employee productivity (Khaksar et al., 2020). Through observation, interaction, and feedback in employee volunteering, employees can integrate knowledge from different domains and apply it to changing work demands, thereby enhancing their problem-solving ability and adaptability for career sustainability (Baartman and De Bruijn, 2011; Godemann, 2008; Khaksar et al., 2020).

Furthermore, by continuously accumulating and integrating knowledge and experience from different fields, employees can enhance team effectiveness and innovation capabilities (Mehta and Mehta, 2018). In volunteering, employees may face challenges different from those in their daily work (Gatignon, 2022), prompting them to approach problems from a fresh perspective. This improvement in innovative capabilities allows them to maintain competitiveness in the workplace and sustain long-term career development.

Based on the theoretical framework and empirical evidence presented, we propose the following hypotheses:

*H2*: Knowledge integration positively influences career sustainability.

*H3*: Knowledge integration mediates the positive relationship between employee volunteering and career sustainability.

### The moderating role of competitive orientation

Competitive orientation typically refers to individuals’ attitudinal and behavioral tendencies toward competition. Specifically, it reflects individuals’ enthusiasm and preference for workplace competition, as well as their pursuit of personal achievement (Chen et al., 2011). In environments characterized by high competitive orientation, individuals tend to focus more on gaining advantages through comparison with others. This intense competitive mindset may sometimes lead to the neglect of teamwork and the importance of resource sharing.

The benefits of competitive orientation have been widely discussed in organizational behavior. Numerous studies have identified significant relationships between competition and individual performance, capabilities, and work outcomes (Wang et al., 2018). However, competitive orientation also carries potential negative implications (Murayama and Elliot, 2012). Moreover, in mixed-motive business environments where individuals face conflicts between personal and collective interest maximization, Chinese individuals tend to make more competitive decisions compared to their “individualistic” Australian counterparts (Chen et al., 2011; Triandis, 2001). Within organizational contexts, employees gain exposure to diverse knowledge domains through volunteering activities and achieve knowledge integration through collaboration. Nevertheless, employees with stronger competitive orientations may prioritize personal achievement and rivalry over cooperation and knowledge sharing. In highly competitive environments, employees might choose to withhold valuable information rather than share it with others, thereby impeding team-level knowledge integration capabilities (Zhang Y. et al., 2022).

Social learning theory also emphasizes that individual learning depends not only on successful experiences but also on feedback and reflection from failures (Bandura, 1985). In highly competitive environments, employees often fear failure and have a low tolerance for it (Jones et al., 2017). Because they are more focused on competing with others, employees may avoid situations that require them to acknowledge their shortcomings and learn from them, which can hinder their willingness and ability to learn. In such situations, employees’ motivation to learn may be suppressed, leading to a decrease in their ability to integrate knowledge.

Therefore, this study hypothesizes that competitive orientation may negatively moderate the impact of employee volunteering on knowledge integration.

*H4*: Competitive orientation moderates the relationship between employee volunteering and knowledge integration; specifically, this positive relationship is weaker when the level of competitive orientation is higher.

*H5*: Competitive orientation moderates the indirect effect of employee volunteering on career sustainability via knowledge integration; specifically, this indirect relationship is weaker when the level of competitive orientation is high.

**Figure 1:**
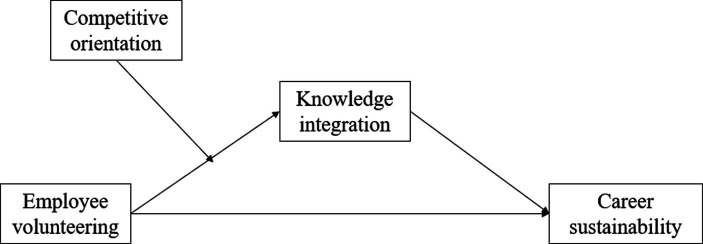
The research model.

The proposed research model is shown in Figure 1.

## Methodology

### Participants and procedures

This study primarily targeted employees from Chinese internet companies, using an online platform for anonymous recruitment. To mitigate common method bias, a three-wave time-lagged data collection approach was implemented, with approximately two-week intervals between each wave. To ensure data quality and maintain the continuity and tracking validity of responses across waves, a unique identification mechanism was established by collecting participants’ Alipay account information through a specially designed questionnaire section. This information was stored separately from the questionnaire responses and was not used in any analysis. After matching, participants were assigned anonymous identification codes, and identifiable information was removed from the analytic dataset. Before completing the online survey, participants were informed of the study purpose, voluntary participation, confidentiality, and data protection procedures, and only those who provided online consent proceeded to the questionnaire. Additionally, an attention-check question was incorporated into each online survey to verify response validity, with data from participants failing this check being excluded from subsequent analyses. The demographic characteristics of the final sample are presented in Table 1.

**Table 1:** Descriptive statistical analysis results.

Variable	Category	Number	Percentage
Gender	Male	152	51.9%
	Female	141	48.1%
Age	≤25 years	25	8.4%
	26–35 years	188	64.3%
	36–45 years	68	23.1%
	≥46 years	12	4.2%
Education level	Vocational/technical degrees or below	36	12.3%
	Bachelor’s degree	233	79.5%
	Master’s degree	23	7.8%
	Doctoral degree or above	1	0.3%
Firm size	50 or fewer employees	37	12.6%
	51–100 employees	119	40.6%
	101–500 employees	113	38.6%
	501 employees or above	24	8.2%
Job position	Staff	160	54.6%
	Front-line manager	97	33.1%
	Middle manager	35	11.9%
	Senior manager	1	0.3%
Job category	Technical R&D	75	25.6%
	Product and design	73	24.9%
	Marketing and operations	94	32.1%
	Business development	22	7.5%
	Support	29	9.9%

Source: Own elaboration.

During the specific data collection phase, at time 1 (T1), we collected participants’ demographic information (such as age, gender and educational attainment) and measured employee volunteering, and 1,208 questionnaires were collected. At time 2 (T2), participants who provided valid responses at T1 were followed up at T2 to measure knowledge integration, and 801 questionnaires were collected. At time 3 (T3), the follow-up continued to measure career sustainability and competitive orientation. Competitive orientation was measured at T3 because it was conceptualized as a relatively stable individual tendency rather than a temporary state (Chen et al., 2011; Reese et al., 2022; Rong et al., 2022). In addition, measuring competitive orientation at an earlier wave could have primed competition-related cognition before the assessment of knowledge integration. This design is also consistent with recommendations to temporally separate key measures to reduce common method bias (Podsakoff et al., 2003). To assess potential attrition bias, we compared the final analytic sample with T1 respondents who did not enter the final sample using available T1 information. We focused on variables that were measured before attrition occurred and were theoretically relevant to the focal model or potentially related to response propensity (Becker et al., 2016; Si et al., 2024). The attrition-bias tests showed that employee volunteering was statistically significant. However, the corresponding effect sizes were small (Cohen’s *d* ≤ 0.200), suggesting that the magnitude of attrition-related differences was limited (Brydges, 2019). The detailed attrition-bias test results are reported in Appendix Table A1.

### Measures

Given that all the items used in this study were originally written in English, we translated these measures into Chinese using a standard back-translation procedure. All items were rated on a five-point Likert scale.

**Employee Volunteering (EV)** was measured using a 5-item scale developed by Rodell (2013), with items such as “I participate in public welfare activities organized by my company.” The scale demonstrated acceptable reliability with a Cronbach’s *α* value of 0.702.

**Knowledge Integration (KI)** was assessed using a 4-item scale adapted from Tiwana (2004), including items like “The knowledge or skills I gained from public welfare activities are highly useful.” The Cronbach’s alpha of the KI scale was 0.649. We further evaluated its measurement quality through additional reliability and validity checks, as reported in Table 2.

**Table 2:** Reliability and validity assessment of the measurement scales.

Construct	Factor loadings	Cronbach’s α	CR	AVE
EV	0.626–0.736	0.702	0.811	0.462
KI	0.606–0.803	0.649	0.793	0.492
CS	0.619–0.767	0.877	0.907	0.496
CPO	0.606–0.768	0.700	0.815	0.469

EV = Employee Volunteering; KI = Knowledge Integration; CPO = Competitive Orientation; CS = Career Sustainability.

**Career Sustainability (CS)** was measured using a 10-item scale by Kong et al. (2023), with items such as “In most ways, my life is close to my ideal,” “I am satisfied with the success I have achieved in my career,” “I am satisfied with the progress I have made in achieving my overall career goals. “The scale exhibited strong reliability with a Cronbach’s α value of 0.877.

**Competitive Orientation (CPO)** was measured using a 5-item scale developed by Chen et al. (2011), with items such as “I feel jealous when others perform better than me at work.” The scale demonstrated good reliability with a Cronbach’s α value of 0.7.

**Control Variables** were selected at both individual and organizational levels. Individual-level controls included gender (1 = Male, 2 = Female), age (How old are you?), and education level (1 = Vocational/technical degrees or below, 2 = Bachelor’s degree, 3 = Master’s degree or above, 4 = Doctoral degrees or above), job position (1 = Staff, 2 = Frontline Manager, 3 = Middle managers, 4 = Senior manager) and job category (1 = Technical R&D category, 2 = Product and design category, 3 = Marketing and operation category, 4 = Business category, 5 = Supporting categories (Human resources, Finance and accounting, Legal Affairs, Administration, etc.)). Organizational-level controls encompassed firm size (1 = 50 people or less, 2 = 51 to 100 people, 3 = 101 to 500 people, 4 = 501 and above), because the size of an enterprise can affect the behavior of employees and the working environment to a certain extent, large-scale enterprises usually have more volunteer service opportunities (Basil et al., 2011).

As shown in Table 2, we further assessed the measurement quality of the study variables in terms of reliability, convergent validity, and discriminant validity. All standardized factor loadings exceeded 0.6, and the AVE values approached the recommended 0.5 threshold (Lam, 2012). In addition, all CR values were above 0.7, indicating acceptable internal consistency reliability. The square root of AVE for each construct exceeded its correlations with the other constructs, supporting satisfactory discriminant validity. In particular, although the Cronbach’s alpha of the KI scale was slightly below the conventional 0.7 threshold, its CR value was 0.793, providing supplementary support for the reliability of this measure. Taken together, these results provide acceptable evidence of convergent validity and internal consistency reliability.

## Results

Table 3 presents the means, standard deviations, and correlation coefficients for the study variables. The correlation analysis revealed significant positive relationships between employee volunteering and both career sustainability and knowledge integration. Furthermore, knowledge integration was significantly and positively correlated with career sustainability. These findings provide a solid foundation for subsequent hypothesis testing.

**Table 3:** Results of variable correlation analysis.

Variable	MEAN	SD	EV	KI	CPO	CS	GEN	AGE	EDU	FS	POS
EV	4.247	0.480	(0.680)								
KI	4.269	0.491	0.510**	(0.701)							
CPO	3.504	0.708	−0.052	0.062	(0.685)						
CS	3.985	0.620	0.351**	0.309**	−0.257**	(0.704)					
GEN	1.48	0.501	−0.002	−0.072	−0.050	−0.071					
AGE	27.31	3.942	0.154**	0.135*	−0.026	0.146*	−0.006				
EDU	1.96	0.463	−0.091	−0.008	0.033	0.003	0.034	0.083			
FS	2.42	0.814	0.149*	0.093	−0.030	0.111	0.003	0.098	0.197**		
POS	1.58	0.710	−0.033	0.003	0.021	0.055	−0.046	0.249**	0.046	0.113	
JC	2.51	1.229	−0.016	0.052	0.066	−0.016	0.300**	0.076	−0.068	−0.108	0.012

EV = Employee Volunteering; KI = Knowledge Integration; CPO = Competitive Orientation; CS = Career Sustainability; GEN = Gender; AGE = Age; EDU = Education level; FS = Firm size; POS = Job position; JC = Job category. Source: Own elaboration.

Although the three-wave time-lagged design helped reduce concerns about common method bias (Podsakoff et al., 2003), we conducted additional statistical checks to further assess its potential influence. First, Harman’s single-factor test showed that the first unrotated factor explained 25.52% of the total variance, which was below the commonly used threshold, suggesting that no single factor dominated the variance structure. Second, we conducted confirmatory factor analysis (CFA) to examine the measurement model. As shown in Table 4, the hypothesized four-factor model demonstrated better fit than the alternative models, indicating satisfactory discriminant validity among the key constructs. Third, we conducted a marker variable test using a theoretically unrelated filler item regarding fruit preference. After controlling for the marker variable, the correlations among the focal constructs remained substantively unchanged, with a maximum change of Δr = 0.021. Taken together, these results suggest that common method bias was unlikely to be a serious threat to the validity of our findings.

**Table 4:** Confirmatory factor analysis results.

Model	χ^2^	df	RMSEA	CFI	TLI	SRMR
EV, KI, CPO, CS	402.849	246	0.047	0.914	0.903	0.051
EV, KI + CPO, CS	531.376	249	0.062	0.844	0.828	0.078
EV, KI + CPO + CS	693.290	251	0.078	0.756	0.732	0.072
EV + KI + CPO + CS	695.988	252	0.078	0.755	0.732	0.072

*N* = 293; CFI = Comparative Fit Index; TLI = Tucker-Lewis Index; SRMR = Standardized Root Mean Square Residual; RMSEA = Root Mean Square Error of Approximation; EV = Employee Volunteering; KI = Knowledge Integration; CPO = Competitive Orientation; CS = Career Sustainability. Source: Own elaboration.

This study employed hierarchical regression analysis to test the theoretical model hypotheses, with results presented in Table 5. According to Model 2 of Table 5, employee volunteering was positively associated with knowledge integration (*b* = 0.517, *p* < 0.001), supporting H1. Furthermore, as shown in Model 5, employee volunteering demonstrated a significant positive effect on career sustainability (*b* = 0.326, *p* < 0.001), while knowledge integration demonstrated a significant positive effect on career sustainability (*b* = 0.206, *p* < 0.01), thus supporting H2.

**Table 5:** Results of hierarchical regression analysis.

Predictor	Knowledge integration			Career sustainability	
	Model 1	Model 2	Model 3	Model 4	Model 5
EV		0.517*** (0.053)	0.251** (0.025)		0.326*** (0.084)
KI					0.206** (0.081)
CPO			0.055* (0.025)		
EV*CPO			−0.077** (0.024)		
GEN	−0.095 (0.060)	−0.097 (0.052)	−0.084 (0.051)	−0.087 (0.076)	−0.070 (0.071)
AGE	0.017* (0.008)	0.006 (0.007)	0.007 (0.007)	0.021* (0.010)	0.011** (0.009)
EDU	−0.030 (0.063)	0.043 (0.055)	0.044 (0.054)	−0.035 (0.080)	0.016 (0.076)
FS	0.060 (0.036)	0.010 (0.032)	0.008 (0.031)	0.079 (0.046)	0.034 (0.043)
POS	−0.031 (0.042)	−0.001 (0.036)	0.007 (0.036)	0.007 (0.053)	0.033 (0.050)
JC	0.032 (0.025)	0.036 (0.021)	0.028 (0.021)	0.002 (0.031)	−0.002 (0.029)
*R* ^2^	0.038	0.277	0.307	0.036	0.160
Adj *R*^2^	0.018	0.259	0.285	0.016	0.136
*F*	1.908	15.585***	13.937***	1.803	6.770***

∗*p* < 0. 05, ∗∗*p* < 0. 01, ∗∗∗*p* < 0. 001. Standard errors are reported in the parentheses.

Second, based on the bootstrapping results, employee volunteering indirectly affects career sustainability through knowledge integration (indirect effect = 0.1065, SE = 0.0442, 95% CI = [0.0243, 0.1995]), supporting H3.

**Figure 2:**
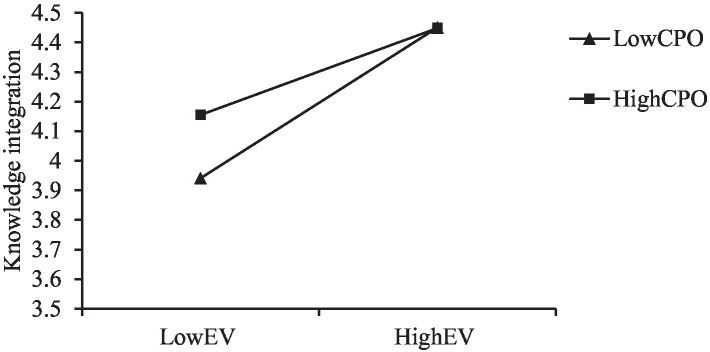
Interaction plot of employee volunteering (EV) and competitive orientation (CPO) regarding predicting knowledge integration.

**Figure 3:**
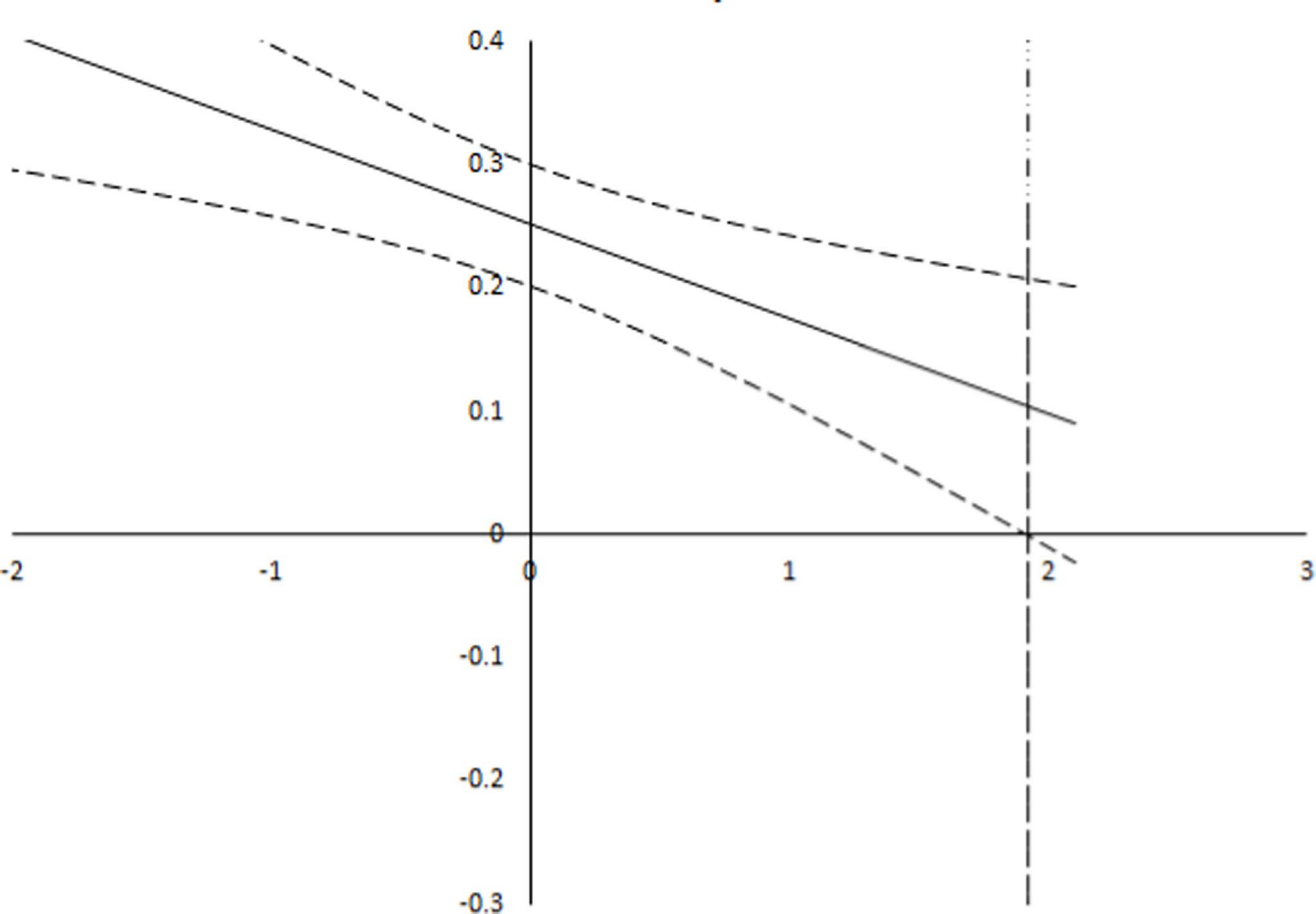
Johnson-Neyman chart.

Finally, H4 proposed that competitive orientation negatively moderates the path from employee volunteering to knowledge integration. As indicated in Model 3 of Table 5, the interaction term between employee volunteering and competitive orientation showed a negative effect on knowledge integration (*b* = −0.077, *p* < 0.01), demonstrating that competitive orientation negatively moderates the employee volunteering - knowledge integration relationship. To directly observe this moderating effect, we plotted the impact of employee volunteering on knowledge integration at different levels of competitive orientation. As shown in Figure 2, the positive effect of employee volunteering on knowledge integration was stronger at low levels of competitive orientation and weaker at high levels of competitive orientation, indicating that competitive orientation weakened this relationship. To further specify the form of this moderating effect, we used the Johnson–Neyman technique to identify the confidence band of the simple slope as shown in Figure 3. When standardized competitive orientation was below 1.9189, the 95% confidence interval of the simple slope excluded 0, indicating a significant moderating effect. Thus, H4 was supported.

H5 proposed that competitive orientation would moderate the indirect effect of employee volunteering on career sustainability through knowledge integration. To examine this moderating effect, we conducted a moderated mediation analysis using the PROCESS macro. As presented in Table 6, the results revealed a significant conditional indirect effect of employee volunteering on career sustainability through knowledge integration at low levels of CPO (effect = 0.1312, SE = 0.0568, 95% CI = [0.0256, 0.2516]). However, this positive effect gradually diminished as competitive orientation increased. At moderate levels of competitive orientation the indirect effect remained significant but decreased to 0.1127 (SE = 0.0484, 95% CI = [0.0216, 0.2161]). When competitive orientation reached high levels, the effect further attenuated to 0.0756 (SE = 0.0364, 95% CI = [0.0147, 0.1594]). Importantly, the index of moderated mediation was statistically significant (index = −0.0093, SE = 0.0056, 95% CI = [−0.0215, −0.0002]), indicating that the strength of the indirect effect systematically varied across different levels of competitive orientation. These findings support H5, indicating that higher competitive orientation weakens the indirect effect of employee volunteering on career sustainability through knowledge integration.

**Table 6:** Results of the moderated mediation analyses.

Indirect pathway	Competitive orientation	Indirect effect	Boot SE	Boot LLCI	Boot ULCI
Employee volunteering → knowledge integration → career sustainability	Low (−1 SD)	0.1312	0.0568	0.0256	0.2516
	Medium (0)	0.1127	0.0484	0.0216	0.2161
	High (+1 SD)	0.0756	0.0364	0.0147	0.1594
	Index	−0.0093	0.0056	−0.0215	−0.0002

Source: Own elaboration.

We further conducted an additional latent-variable structural equation modeling (SEM) analysis to assess the robustness of our findings, and the results, reported in Appendix Table A2, provided further support for our conclusions.

## Discussion

This study examined the impact of employee volunteering on career sustainability, emphasizing the mediating role of knowledge integration and the moderating effect of competitive orientation. Drawing on social learning theory, this study tested the proposed hypotheses using three-wave survey data from 293 employees. The findings show that employee volunteering promotes career sustainability through knowledge integration. Competitive orientation weakens the positive relationship between employee volunteering and knowledge integration, thereby attenuating the indirect effect of employee volunteering on career sustainability through knowledge integration.

### Theoretical implications

Drawing on social learning theory, this study unveils the mechanism linking employee volunteering to career sustainability, offering new directions for theoretical development in the field of career studies.

Firstly, this study enriches the research on career sustainability and employee volunteering. Although existing literature has mainly focused on the impact of social responsibility practices at the organizational level or personal characteristics and proactive personalities at the individual level on career sustainability (Aguinis and Glavas, 2012; De Roeck and Maon, 2018; Lent et al., 2024), it has overlooked the positive impact of employee volunteering behavior, an individual employee behavior (Rodell et al., 2016), on career sustainability. Existing studies have verified the positive effect of employee volunteering on job satisfaction (Boezeman and Ellemers, 2009; Rodell, 2013), and job satisfaction is precisely an important component of career sustainability (De Vos et al., 2020; Schweitzer et al., 2023). Based on this, this study incorporates career sustainability into the analytical framework of employee volunteering. Our findings suggest that employee volunteering promotes career sustainability by enhancing knowledge integration, thereby enriching our understanding of the outcomes of employee volunteering and the antecedents of career sustainability.

Second, by introducing knowledge integration as a mediating variable, the study deepens the understanding of the mechanisms behind career sustainability. Previous research has highlighted the importance of individual competencies (Zhang W. et al., 2022) and organizational training (Bozionelos et al., 2020) in career sustainability. This study further enriches the research on individual competencies, revealing how knowledge integration effectively promotes career sustainability. Additionally, existing studies have demonstrated the positive impact of cross-disciplinary knowledge and experience gained through volunteering on work performance, innovation capability, and workplace adaptability (Caligiuri et al., 2019; Pless et al., 2011). This study further validates the positive effects of employee volunteering and knowledge integration, emphasizing the role of employee volunteering in enhancing career sustainability through the improvement of knowledge integration.

Furthermore, the moderating role of competitive orientation reveals that excessive competition can reduce the benefits of volunteerism, such as knowledge integration. These findings qualify and extend existing research on competitive orientation by showing that competitive orientation may serve as a boundary condition that weakens the learning benefits of employee volunteering. Although competition is often seen as a motivator (Kalra et al., 2021; Schrock et al., 2016; Wang et al., 2018), some scholars have highlighted its negative effects. A strong competitive orientation creates uncertainty and concern over outcomes, becoming a major source of work stress (Fletcher et al., 2008; Keller et al., 2016) and interpersonal conflict (Boz Semerci, 2019). In highly competitive settings, employees may focus on personal interests over team goals, undermining cohesion and reducing organizational performance (Hernaus et al., 2019). This study extends existing research on the behavioral impact of competitive orientation and offers a nuanced theoretical model for understanding how individual dispositions interact with work environments to shape career development.

### Managerial implications

Building upon these theoretical contributions, this study offers several important practical implications. First, organizations can institutionalize employee volunteering programs through initiatives such as cross-departmental collaborations or skill-based volunteering to enhance employees’ knowledge integration capabilities, thereby strengthening their career adaptability and long-term development potential.

In addition, this study underscores the necessity of balancing competitive cultures: managers should be vigilant about the inhibitory effects of excessive competition on knowledge sharing. This can be mitigated through team goal setting, collaborative incentive mechanisms, or inclusive leadership styles to alleviate the negative impacts of competitive orientation.

Furthermore, this study provides empirical support for synergistic approaches between corporate social responsibility and employee development: transforming volunteering from a unidirectional “image-building” exercise into a vehicle for employee capability enhancement not only reinforces the organization’s social value but also creates a win-win scenario for both employee career growth and organizational performance. These findings offer actionable insights for organizations to develop sustainable talent strategies in rapidly changing business environments.

## Limitations and directions for future research

While this study has explored the relationships among employee volunteering, knowledge integration, competitive orientation, and career sustainability, yielding significant findings, several limitations should be acknowledged. First, the data primarily came from employees in Chinese internet companies, and the geographical and industrial characteristics of the sample may limit the generalizability of the findings. In addition, the sample was relatively concentrated among younger employees and bachelor’s-degree holders, which should be considered when interpreting the results. Future research could expand the sample to different regions, industries, age groups, and educational backgrounds to further examine the robustness and boundary conditions of the proposed model.

Second, the data were mainly collected through questionnaires, which may be subject to social desirability bias and recall bias. Employees might tend to report their positive volunteering behaviors and career satisfaction, potentially affecting data accuracy. Future studies could consider incorporating multiple data sources, such as peer or supervisor evaluations, to mitigate this limitation.

Third, the competitive orientation variable in this study was measured primarily through a single dimension, which may not fully capture the diverse competitive psychology and behaviors in the workplace. Future research could further explore the multidimensional structure of competitive orientation, such as internal versus external competition and cooperative competition, to provide a more comprehensive understanding of its impact on career sustainability. Although competitive orientation was conceptualized as a relatively stable individual tendency, it was measured only at Time 3 in this study. Future research could measure competitive orientation at baseline or repeatedly across waves to directly examine its temporal stability.

Finally, future studies should also focus on the possible differential effects of different types of volunteer services. Formal volunteer services and informal, intra-organizational and inter-organizational volunteer behaviors may have different impacts on knowledge integration and career sustainability. An in-depth exploration of these differences will help clarify how employee volunteering can better support career development. Although certain limitations remain, this study offers new insights into the relationships among employee volunteering, knowledge integration, competitive orientation, and career sustainability.

## Data Availability

The raw data supporting the conclusions of this article will be made available by the authors, without undue reservation.
