# Supplementary material for: Employee volunteering, knowledge integration, and career sustainability: the moderating role of competitive orientation
Source: Front Psychol. 2026 Jul 28;17:1876278. doi: 10.3389/fpsyg.2026.1876278 (PMC13457096; doi:10.3389/fpsyg.2026.1876278)
Supplement: Supplementary file 1 [file Table_1.docx]

Appendix Table A1. Attrition Bias Tests

| T-test | | | | |
| --- | --- | --- | --- | --- |
| Variable | t | df | p | Cohen's d |
| EV | 2.280 | 593.800 | 0.023 | 0.138 |
| AGE | -0.530 | 590.300 | 0.596 | -0.032 |
| Chi-square tests | | | | |
| Variable | χ² | df | p | Cramer's V |
| EDU | 5.122 | 3 | 0.163 | 0.065 |
| JC | 8.994 | 4 | 0.061 | 0.086 |

Notes: EV = Employee Volunteering; AGE = Age; EDU = Education Level; JC = Job Category.

Appendix Table A2. Latent Moderated SEM Robustness Check

| χ² | df | χ²/df | CFI | TLI | RMSEA |
| --- | --- | --- | --- | --- | --- |
| 144.559 | 83 | 1.742 | 0.942 | 0.926 | 0.05 |
|  | β | SE | p | 95% CI | |
| EV→KI | 0.763 | 0.088 | <0.001 | [0.544, 0.888] | |
| EV × CPO→KI | -0.202 | 0.180 | 0.031 | [-0.741, -0.035] | |
| KI→CS | 0.470 | 0.108 | <0.001 | [0.441, 0.865] | |

Note: EV = Employee Volunteering; KI = Knowledge Integration; CPO = Competitive Orientation; CS = Career Sustainability.
